# Polyethylene Glycol Electrolyte Lavage Solution versus Colonic Hydrotherapy for Bowel Preparation before Colonoscopy: A Single Center, Randomized, and Controlled Study

**DOI:** 10.1155/2014/541586

**Published:** 2014-06-05

**Authors:** Yan Cao, Kai-Yuan Zhang, Jiao Li, Hao Lu, Wan-Ling Xie, Sheng-Tao Liao, Dong-Feng Chen, Deng-Feng Zeng, Chun-Hui Lan

**Affiliations:** ^1^Department of Gastroenterology, Daping Hospital, The Third Military Medical University, 10 Changjiang Branch Road, Chongqing 400042, China; ^2^Cadet Brigade, The Third Military Medical University, 30 Gaotanyan Street, Chongqing 400038, China; ^3^Nursing Department, Daping Hospital, The Third Military Medical University, 10 Changjiang Branch Road, Chongqing 400042, China

## Abstract

This single center, randomized, and controlled study aimed to compare the effectiveness and safety of polyethylene glycol electrolyte lavage (PEG-EL) solution and colonic hydrotherapy (CHT) for bowel preparation before colonoscopy. A total of 196 eligible outpatients scheduled for diagnostic colonoscopy were randomly assigned to the PEG-EL (*n* = 102) or CHT (*n* = 94) groups. Primary outcome measures included colonic cleanliness and adverse effects. Secondary outcome measures were patient satisfaction and preference, colonoscopic findings, ileocecal arrival rate, examiner satisfaction, and cecal intubation time. The results show that PEG-EL group was associated with significantly better colonic cleanliness than CHT group, fewer adverse effects, and increased examiner satisfaction. However, the CHT group had higher patient satisfaction and higher diverticulosis detection rates. Moreover, the results showed the same ileocecal arrival rate and patient preference between the two groups (*P* > 0.05). These findings indicate that PEG-EL is the preferred option in patients who followed the preparation instructions completely.

## 1. Introduction

China has a high incidence of lower gastrointestinal (GI) malignancies, including colon and rectal cancer [1]. Colonoscopy is a minimally invasive procedure and is currently the standard method to assess colonic mucosa [2]. It is typically ordered to exclude or identify early malignant diseases in patients with warning symptoms [3]. It has been widely used in patients with intestinal disorders to diagnose or treat colon diseases, as well as for other interventions. However, because colonoscopy is predominantly used as a screening tool for lower GI disorders, patient tolerability and compliance are critical for its effectiveness [3]. Therefore, comfortable and well-tolerated bowel preparation is essential for effective and accurate colonoscopy examinations [4]. Attempts to improve patient acceptance of bowel preparations have primarily centered on changing the laxatives by altering electrolyte content or adding flavoring [5], or changing the bowel preparation method, such as colonic hydrotherapy [6].

Since 1980, polyethylene glycol-electrolyte lavage (PEG-EL) has become one of the most commonly used laxatives for preparation before colonoscopy [7]. Compared to PEG-EL, the sodium phosphate (NaP) method has similar, or perhaps better, efficacy and requires smaller fluid intake volumes [8]. However, NaP can potentially cause electrolyte imbalance, particularly hypernatremia and hyperphosphatemia, which could result in renal failure [9]. Recently, PEG-EL has become the most recommended laxative due to its proven safety and efficacy [10]. However, PEG-EL is hard for some patients to tolerate due to its severe adverse effects such as nausea, vomiting, and abdominal distension [11]. Moreover, there is a congestive heart failure risk, particularly for patients who have chronic renal insufficiency or left cardiac insufficiency [12]. Colonic hydrotherapy (CHT), which has been used as a constipation treatment [13] as well as for bowel preparation before colectomy [14], is currently being evaluated as a new preparation method before colonoscopy. It has been reported that CHT has advantages of shorter operating times, lower incidences of adverse effects, and higher colonic cleanliness for colectomy preparation [14]. Previous studies in China demonstrated that CHT was superior to PEG-EL for colonoscopic preparation [15, 16].

Therefore, this study aimed to compare the effectiveness and safety between PEG-EL and CHT in bowel preparation for colonoscopy. We assessed colonic cleanliness, adverse effects, cecal intubation time, ileocecal arrival rate, colonoscopic findings, examiner satisfaction, and patient satisfaction.

## 2. Materials and Methods

### 2.1. Study Design

This study was a single-center, single-blinded, prospective, randomized, and controlled trial. The colonoscopists performing the examination were unaware of the preparation regimen used by the patient. Randomization was performed according to the table of random numbers. The Institutional Review Board and Institutional Research Ethics Committee at Daping Hospital, the Third Military Medical University, approved the study protocol, which was in accordance with the latest version of The Declaration of Helsinkifor human experimentation (China Clinical Trial Registry no. ChiCTR-OCS-13003526). All subjects provided written informed consent prior to enrollment. After referral by general practitioners or gastroenterologists for lower GI symptoms, consecutive patients of either sex (*n* = 320) presenting to our outpatient gastroenterology clinics were scheduled for diagnostic colonoscopy between May 2012 and May 2013. Inclusion criteria included (1) being aged 18 to 70 years; (2) being able to respond to self-administered questionnaires; and (3) suitability for bowel preparation. Exclusion criteria included (1) previous history of colon surgery; (2) pregnancy or lactation; (3) psychiatric conditions and inability to provide informed consent; (4) uncontrolled hypertension; and (5) unstable diabetes mellitus, serious hepatic or renal dysfunction, or cardiopulmonary compromise. After initial screening and providing informed consent, a total of 206 eligible patients were randomly assigned to the PEG-EL (*n* = 102) or CHT (*n* = 104) groups. Each patient received appropriate written preparation instructions. Although some patients had different degrees of adverse effects, all patients followed the preparation instructions completely.

## 3. Colonoscopic Preparation Instructions

All patients had electrocardiography, routine blood tests, and coagulation tests prior to colonoscopic examination. Patients in both groups were instructed to consume a clear liquid diet on the evening before the colonoscopy. They were also forbidden to eat the morning of the procedure and to drink 2 h before the examination. Patients in the PEG-EL group began consumption of the PEG-EL laxative (137.15 g in 2 L of water) at 5:00 am before the colonoscopy. Patients were instructed to consume 200 mL every 10 min; thus, before 7:00 am, the last dose of PEG-EL was consumed. For patients of CHT group, a JS-308-f colonic dialysis machine (Jinjian Medical Equipment Co. Ltd., Guangzhou, China) was used for colonic hydrotherapy for 40 to 60 min before colonoscopy. During this process, water pressure was 20 kPa, water temperature was 38°C, and the perfusion flow was 600 mL/min in a pulse-like manner. Water was infused at a rate of 800–2000 mL per time based on the tolerance of patients. The infused times were 5–8 until the liquid washed out became transparent. Moreover, nursing care, including psychology and abdominal massage, was essential for helping the patients cooperate. The bowel preparation procedures are as follows. (1) Patient preparation: scientific and complete urination and defecation are critical. Patients are placed in a comfortable position, and the purpose and CHT methods were explained in a proper manner. (2) Standard operating procedures: Vaseline cream is applied to the anal canal for lubrication and the colonic dialysis machine parameters are adjusted. Patients were instructed to breathe deeply to relax the anal canal before insertion. The drainage pipe is connected and electronic pliers are inserted in a soft manner. The water is in a pulse-like style. The condition of the patients was closely observed during the procedure. The above steps were repeated until washed out liquid became transparent. (3) Tube withdrawal procedure: patients were instructed to clamp the anus tightly. The operator holds gauze in the left hand and quickly and gently withdraws the tube with the right hand. Vaseline cream is again applied to the anal canal for lubrication. Patients were instructed to increase their walking and to defecate as much as possible to expel the liquid.

In the morning at exactly 8:30 am, patients were placed in the left lateral decubitus position throughout the procedure and were continuously monitored by an assistant colonoscopist or colonoscopy nurse using an electronic multifunctional patient monitor unit (Mindray Medical, Shenzhen, China). Systolic and diastolic blood pressure (SBP/DBP), pulse rate (PR), and oxygen saturation based on pulse oximetry (SpO_2_) were monitored. Anesthesia was performed in all patients. Additional intravenous access was established, and nasal oxygen was administered at a flow rate of 2 L/min. An independent anesthesiologist administered a mixture of 0.01-0.02 mg/kg midazolam (Nhwa Pharmaceutical Group Co., Xuzhou, China), 0.4 *μ*g/kg remifentanil (Renfu Pharmaceutical Co., Yichang, China), and 1-2 mg/kg propofol (AstraZeneca, Caponago, Italy). Patients were maintained at a Ramsay sedation scale (RSS) score greater than 4 throughout the colonoscopy procedure. These procedures took approximately 30 min; therefore, the delay between the last preparation dose and the colonoscopy procedure is approximately 2 hours, which is sufficient for PEG-EL colonoscopic preparation.

After that colonoscopy procedures were conducted using standard adult video colonoscopes (CF-Q260AL; Olympus Co. Ltd., Tokyo, Japan) by an independent team of board-certified colonoscopists who were blinded to the preparation regimen. Patients were instructed not to discuss their colon cleansing preparation with the colonoscopists, either before or during the procedure. All colonoscopies were performed using the one-man method.

Patients were delivered to the recovery unit after the colonoscopic examination and were not discharged from the clinic until they regained consciousness. All patients were advised to eat only cold soft food at least 2 h after the procedure. It was also recommended that patients do not drive or sign legal documents within 24 h after the examination.

## 4. Data Collection

At the end of the procedure, endoscopists classified the quality of colon cleansing as: Grade 1, “Excellent”: a small volume of clear liquid and no stool; Grade 2, “Good”: a large volume of clear liquid or some semisolid stool that could be washed or removed by suction (some suctioning required, no limitations); Grade 3, “Poor”: semisolid stool that could not be washed or removed by suction (thus, small lesions may be missed); Grade 4, “Failed”: having solid stools and hard to observe, thereby requiring repreparation and another colonoscopy. The preparation was unqualified if the colonic cleanliness score was Grade 3 or 4. The patient with the Grade 4 score was recommended to repeat the bowel preparation. In addition, examiner satisfaction was rated as (1) for extremely unsatisfied, (2) for moderately unsatisfied, (3) for neither satisfied nor dissatisfied, (4) for moderately satisfied, and (5) for extremely satisfied. Other outcome measures were also recorded, including cecal intubation time (from intubation to withdrawal), ileocecal arrival rate (colonoscope arrival at the ileocecal), and colonoscopic findings. In particular, all endoscopists participating in this study were well trained on these scales, and two endoscopists independently evaluated and recorded the scores as part of blinded study. Differences were resolved through discussion with another endoscopist (Lan).

All patients who successfully underwent their assigned colonoscopy procedure were asked to answer a self-administered questionnaire to acquire baseline characteristics. Compliance with the preparation instructions, the prevalence of adverse effects associated with the preparation, and patient satisfaction and preference were determined. Baseline characteristics of patients included age, sex, body mass index (BMI), constipation history, previous colonoscopy experience, alcohol consumption, and smoking status. Consumption of alcohol was classified as (1) for little or no drinking (alcohol < 50 g/day) or (2) for drinking (alcohol ≥ 50 g/day). Tobacco use was described as (0) for no smoking, (1) for smoking <20 cigarettes/day, or (2) for ≥20 cigarettes/day. Finally, patient satisfaction was defined as (1) for extremely unsatisfied, (2) for moderately unsatisfied, (3) for neither satisfied nor dissatisfied, (4) for moderately satisfied, and (5) for extremely satisfied. Patient preference for their assigned colonoscopy was assessed relative to their bowel preparation method.

## 5. Statistical Analyses

Quantitative data are expressed as the mean ± standard deviation (SD) and compared using independent *t*-tests. All qualitative data are expressed as *n* (%) and compared using *χ* ^2^ tests. Fisher's exact tests were used for correction if necessary. All *P* values were 2-tailed and *P* values less than 0.05 were considered statistically significant.

## 6. Results

### 6.1. Baseline Characteristics of the Patients

**Figure 1:**
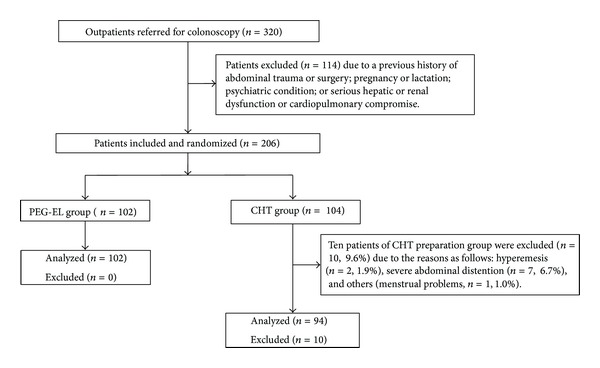
Flow chart of patient disposition and assignment to different patient populations.

Patients from the two groups followed all instructions regarding the dietary restrictions before the colonoscopy. Ten patients in the CHT group (9.6%) gave up the bowel preparation and colonoscopy because of hyperemesis (*n* = 2, 1.9%), severe abdominal distention (*n* = 7, 6.7%), and menstrual problems (*n* = 1, 1.0%). Therefore, 196 patients were included in the PEG-EL (*n* = 102) and CHT (*n* = 94) groups (Figure 1). Patients in the two groups were comparable in baseline characteristics, including age, sex, BMI, underlying constipation, alcohol intake levels, smoking status, and previous experience with bowel preparations. As shown in Table 1, both groups of patients had similar baseline characteristics and reasons for requiring colonoscopies (*P* > 0.05). Therefore, it was statistically acceptable to make comparisons between these two groups.

## 7. Bowel Preparation Quality and Colonoscopic Adverse Effects

**Figure 2:**
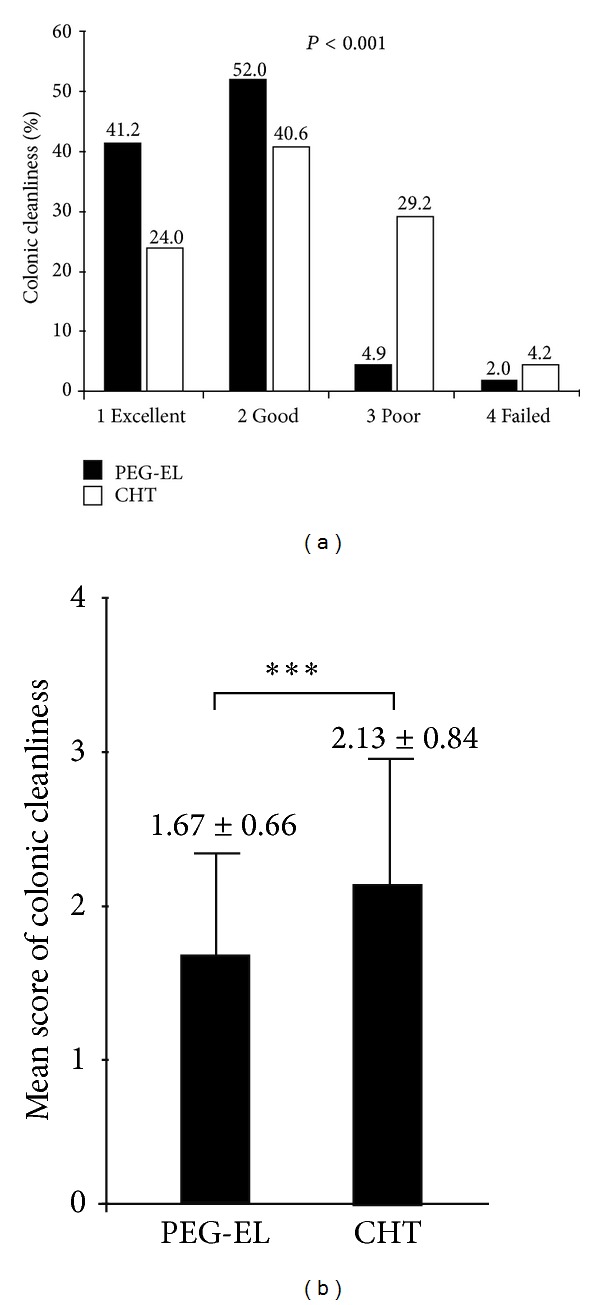
Bowel preparation quality (%, (a)) and mean scores (mean ± SD, (b)) in the PEG-EL laxative group and CHT group were graded by colonoscopists (*P* < 0.001).

Colonoscopists assessed the quality of bowel cleansing, and the mean quality scores are shown in Figure 2. The mean score was 1.67 ± 0.66 in the PEG-EL group (*n* = 102), whereas in patients who completed the CHT preparation (*n* = 94), the mean score was 2.13 ± 0.84. The bowel preparation quality was significantly better in the PEG-EL group than in the CHT group (*P* < 0.001).

Adverse effects are shown in Table 2. Adverse effects were significantly more frequent in the CHT preparation group compared to the PEG-EL group (CHT versus PEG-EL, 40.4% versus 4.9%, *P* < 0.05). For some specific adverse effects (i.e., nausea, vomiting, dizziness, palpitations, and headache), there were no significant differences in incidence between the groups. However, as shown in Table 2, abdominal distension and abdominal cramps were significantly more frequent in patients using the CHT preparation compared to patients using the PEG-EL preparation (for abdominal distension, CHT versus PEG-EL, 38.3% versus 2.0%, *P* < 0.05; for abdominal cramps, CHT versus PEG-EL, 4.3% versus 0%, *P* < 0.05). Thus, the CHT preparation resulted in a significantly higher incidence of adverse effects (CHT versus PEG-EL, 40.4% versus 4.9%, *P* < 0.05).

### 7.1. Cecal Intubation Time, Ileocecal Arrival Rate, and Colonoscopic Findings

There were no statistical differences in cecal intubation time between the groups (CHT versus PEG-EL, 14.85 ± 8.58 versus 15.86 ± 10.60 min, *P* > 0.05). The ileocecal arrival rate was similar for both groups (CHT versus PEG-EL, 94.7% versus 95.1%, *P* > 0.05). Regarding the colonoscopic findings, there were no significant differences for detection rates of inflammation, polyps, and tumors (*P* > 0.05). However, CHT had a higher detection rate of diverticulosis compared to PEG-EL (CHT versus PEG-EL, 14.9% versus 1.0%, *P* < 0.05) (Table 3).

### 7.2. Examiner Satisfaction, Patient Satisfaction, and Preference

**Figure 3:**
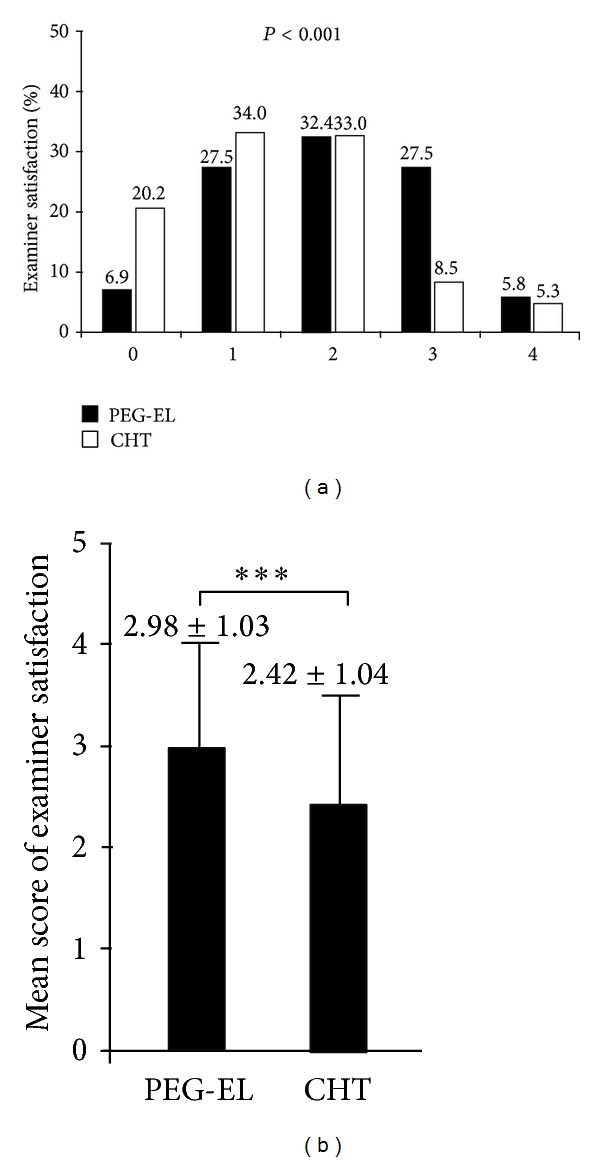
Examiner satisfaction (%, (a)) and mean scores (mean ± SD, (b)) for the assigned colonoscopy in the PEG-EL laxative and CHT groups (*P* < 0.001).

The mean scores of examiner satisfaction are shown in Figure 3. The mean score was 2.98 ± 1.03 in the PEG-EL group (*n* = 102), whereas in patients who completed the CHT preparation (*n* = 96), the mean score was 2.42 ± 1.04. The mean scores for examiner satisfaction for the PEG-EL group and CHT group were statistically different (*P* < 0.001).

**Figure 4:**
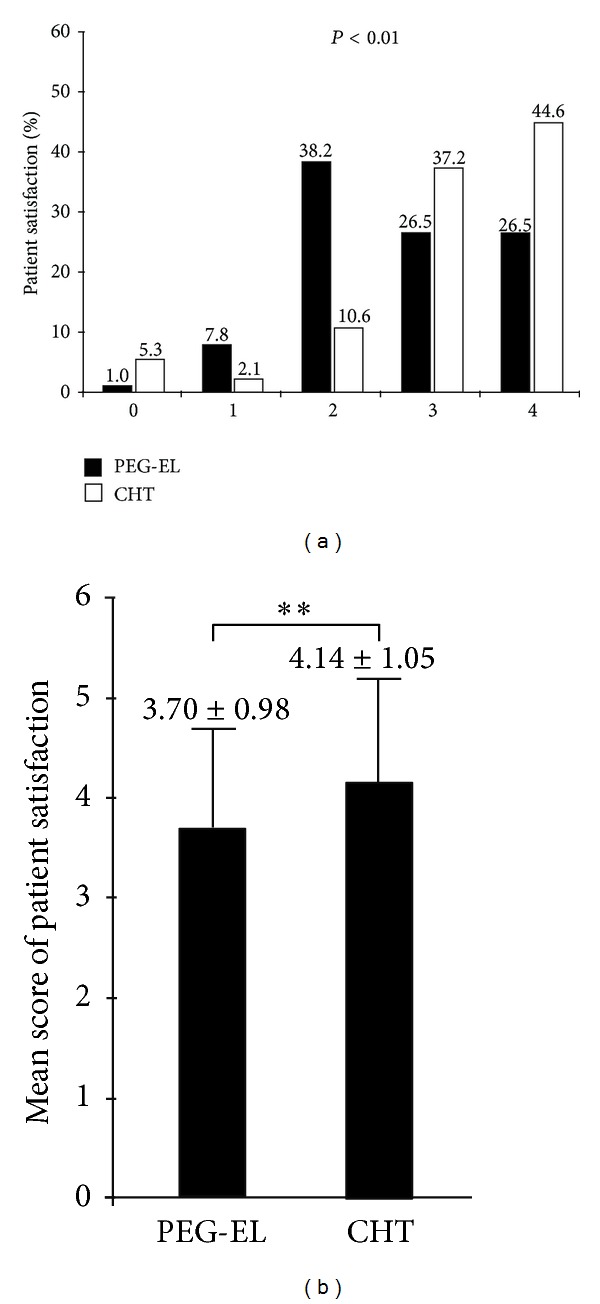
Patient satisfaction (%, (a)) and mean scores (mean ± SD, (b)) for the assigned colonoscopy in the PEG-EL laxative and CHT groups (*P* < 0.01).

**Figure 5:**
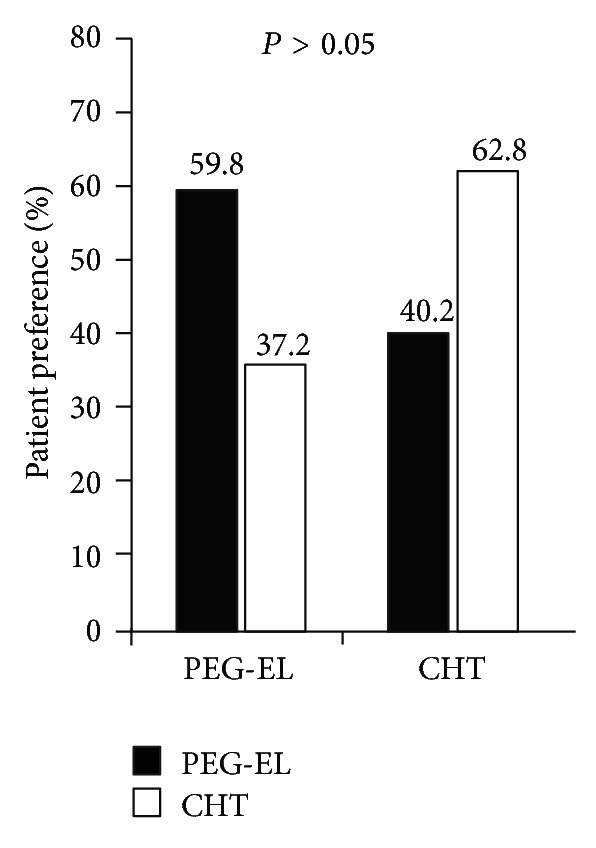
Patient preference (%) for the assigned colonoscopy between the PEG-EL laxative and CHT groups (*P* > 0.05).

Patients in the CHT preparation group had significantly higher rates of adverse effects compared to the PEG-EL group (CHT versus PEG-EL, 40.4% versus 4.9%, *P* < 0.05). However, they also had significantly higher patient satisfaction levels (CHT versus PEG-EL, 4.14 ± 1.05 versus 3.70 ± 0.98, *P* < 0.01, Figure 4). Furthermore, we rated patient preference in 102 subjects in the PEG-EL group and 94 subjects in the CHT group (Figure 5). The bowel preparation method had significant impact on the patient's preference of colonoscopic preparation method for subsequent colonoscopy examinations: the PEG-EL group (CHT versus PEG-EL, 37.2% versus 59.8%, *P* < 0.01) and the CHT group (CHT versus PEG-EL, 62.8% versus 40.2%, *P* < 0.01). In general, patients tended to prefer the CHT method (PEG-EL versus CHT, 49% versus 51%) for a subsequent examination, although the differences were not significant. Moreover, there were twenty patients (Table 1) who experienced both colonoscopy experiences for bowel preparation, and patients preferred CHT over PEG-EL (CHT versus PEG-EL, 12 patients (60%) versus 8 patients (40%)) for a subsequent examination.

## 8. Discussion

This study was designed to compare the effectiveness and safety of PEG-EL and CHT in bowel preparation for colonoscopy. Our results indicate that PEG-EL resulted in better colonic cleanliness, fewer adverse effects, and higher examiner satisfaction. However, the PEG-EL preparation had lower patient satisfaction and a lower diverticulosis detection rate compared with CHT.

Efficacy indices, such as mean quality scores for bowel cleansing and examiner satisfaction, were found to be significantly superior in the PEG-EL group compared to the CHT group. There were no significant differences in cecal intubation times and ileocecal arrival rates between groups; however, a higher detection rate of diverticulosis was noted in the CHT group. Moreover, the time duration of bowel preparation varies between these two methods, with CHT preparation (less than 1 hour) significantly less than PEG-EL (approximately 4 hours). Therefore, we conclude that PEG-EL is, to some extent, more efficient and efficacious than CHT although the CHT preparation takes less time. This result differs from previous studies because there was no such standardized operation process for CHT. Increased effort should be made to establish scientific CHT procedures. Patient cooperation could be enhanced via reinforcing communication. Therefore, CHT could become a more efficacious and tolerated choice for bowel preparation if there is effective communication with patients, skilled operating practices by nurses, good cooperation from patients, and proper food preparation before the operation. Moreover, for patients, the cost was the same and there was no need for hospitalization with either method of bowel preparation. However, the CHT patients did not have to take any medications. The JS-308-f colonic dialysis machine is the only necessary equipment for CHT and it is convenient to operate. However, for the PEG-EL preparation, no additional equipment is required.

There is currently no standard system to evaluate patient satisfaction and discomfort associated with colonoscopy. Consistent with previous findings, all patients followed the preparation instructions completely; however, CHT was associated with higher rates of adverse effects and failure compared with PEG-EL (*P* < 0.05). Moreover, in light of the 10 patients from the CHT group who gave up the bowel preparation and colonoscopy, it can be assumed that the CHT group had an even higher rate of adverse effects compared to the PEG-EL group. These 10 patients would likely choose PEG-EL for any future colonoscopies. Thus, we conclude that CHT was less tolerated than PEG-EL. Interestingly, there were significantly higher mean scores of patient satisfaction in the CHT group. Patients in the CHT group subjectively had less discomfort. The bowel preparation method significantly impacted patient preferences in choosing a colonoscopic preparation method for subsequent examinations. We found that patients in the CHT group had higher satisfaction rates than those in the PEG-EL group. These data suggest that CHT could be applied after improvement, although some side effects remain. However, it has a higher satisfaction in the bearable patients.

There are several limitations in this study. First, it would be preferable to compare previous and current bowel preparation experiences in the same patient to assess patient satisfaction and preference. Second, we did not systematically measure serum electrolytes in either method. Therefore, we have no information on treatment-induced electrolyte changes. Third, the same operator should conduct the CHT procedures because operating proficiency may significantly impact the results. Fourth, patient discomfort resulting from both methods should be further investigated, including their influence on sleep and diet.

Bowel preparation using PEG-EL is associated with better bowel cleansing, examiner satisfaction, and fewer adverse effects compared with CHT. These data indicate that PEG-EL should be the preferred option in patients who followed the bowel preparation instructions completely.

## Figures and Tables

**Table 1:** Baseline characteristics of patients (*n* = 196).

	PEG-EL (*n* = 102)	CHT (*n* = 94)	*P*
Age (years)	47.52 ± 12.12	46.90 ± 10.95	0.71
Range	20–78	18–68	
Sex			0.919
Male	55 (53.9%)	50 (53.2%)	
Female	47 (46.1%)	44 (46.8%)	
BMI	22.14 ± 2.22	22.72 ± 3.20	0.145
Astriction condition	24 (23.53%)	32 (32.98%)	0.141
Previous colonoscopy experience			0.182
None	88 (86.3%)	72 (76.6%)	
PEG-EL	13 (12.7%)	19 (20.2%)	
CHT	1 (0.9%)	3 (3.2%)	
Consumption of tobacco			0.518
0 (no smoking)	78 (76.5%)	72 (76.6%)	
1 (<20 cigarettes/day)	16 (15.7%)	11 (11.7%)	
2 (≥20 cigarettes/day)	8 (7.8%)	11 (11.7%)	
Consumption of alcohol			0.419
1 (alcohol < 50 g/day)	99 (97.1%)	88 (93.6%)	
2 (alcohol ≥ 50 g/day)	3 (2.9%)	6 (6.4%)	
Reasons for Colonoscopy			0.301
Screening	24 (23.5%)	23 (24.5%)	
Anemia	0 (0.0%)	1 (1.1%)	
Hematochezia	2 (2.0%)	0 (0.0%)	
Body weight loss	2 (2.0%)	6 (6.4%)	
Abdominal pain or discomfort	37 (36.3%)	36 (38.3%)	
Constipation	6 (5.9%)	7 (7.4%)	
Diarrhea	12 (11.8%)	8 (8.5%)	
Change in bowel habits	13 (12.7%)	12 (12.8%)	
Follow-up after polyp or polypectomy	1 (1.0%)	1 (1.1%)	
Follow-up for inflammatory bowel disease	5 (4.9%)	0 (0.0%)	

Data are presented as the mean ± SD or *n* (%).

**Table 2:** Prevalence of adverse effects.

	PEG-EL (*n* = 102)	CHT (*n* = 94)
Adverse effects	4 (4.9%)*	38 (40.4%)*
Nausea	0 (0.0%)	0 (0.0%)
Vomiting	2 (2.0%)	2 (2.1%)
Abdominal distension	2 (2.0%)*	36 (38.3%)*
Abdominal cramps	0 (0.0%)	4 (4.3%)
Dizziness	1 (1.0%)	1 (1.0%)
Palpitations	0 (0.0%)	0 (0.0%)
Headache	0 (0.0%)	0 (0.0%)

*P < 0.05; data are presented as *n* (%).

**Table 3:** Colonoscopy duration and colonoscopic findings.

	PEG-EL (*n* = 102)	CHT (*n* = 94)
Cecal intubation time (min)	15.86 ± 10.60	14.85 ± 8.58
Ileocecal arrival	97 (95.1%)	89 (94.7%)
Colonoscopic findings		
Inflammation	4 (3.9%)	8 (8.5%)
Polyps	15 (14.7%)	18 (19.1%)
Tumor	0 (0.0%)	3 (3.2%)
Diverticulosis	1 (1.0%)*	14 (14.9%)*

*P < 0.05*;* data are presented as the mean ± SD or *n* (%).
